# Microrchidia CW-Type Zinc Finger 2, a Chromatin Modifier in a Spectrum of Peripheral Neuropathies

**DOI:** 10.3389/fncel.2022.896854

**Published:** 2022-06-03

**Authors:** Arnaud Jacquier, Simon Roubille, Patrick Lomonte, Laurent Schaeffer

**Affiliations:** ^1^INMG-Pathophysiology and Genetics of Neuron and Muscle, CNRS UMR 5261, INSERM U1315, Université Claude Bernard Lyon 1, Faculté de Médecine Lyon Est, Lyon, France; ^2^Hospices Civils de Lyon, Groupement Est, Bron, France

**Keywords:** MORC2, Charcot-Marie-Tooth (CMT) disease, spinal muscular atrophy, DIGFAN syndrome, DNA damage repair (DDR), HUSH complex, transcriptional modulation, epigenetic

## Abstract

*Microrchidia CW-type zinc finger 2 (MORC2)* gene encodes a protein expressed in all tissues and enriched in the brain. MORC2 protein is composed of a catalytic ATPase domain, three coil-coiled domains allowing dimerization or protein complex interaction, a zinc-finger CW domain allowing DNA interaction, and a CHROMO-like (CHRromatin Organization Modifier) domain. Recently, *de novo* or dominantly inherited heterozygous mutations have been associated with a spectrum of disorders affecting the peripheral nervous system such as the Charcot-Marie-Tooth disease, spinal muscular atrophy-like phenotype disorder, or a neurodevelopmental syndrome associated with developmental delay, impaired growth, dysmorphic facies, and axonal neuropathy (DIGFAN). In this review, we detail the various mutations of MORC2 and their consequences on clinical manifestations. Possible genotype-phenotype correlations as well as intra and inter-family variability are discussed. MORC2 molecular functions such as transcriptional modulation, DNA damage repair, and lipid metabolism are then reviewed. We further discuss the impact of MORC2 mutations on the epigenetic landscape in the neuromuscular system and hypothesize probable pathophysiological mechanisms underlying the phenotypic variability observed.

## Introduction

Microrchidia (MORC) protein superfamily is composed of nuclear proteins playing a role in chromatin remodeling and epigenetic silencing that are widely conserved across eukaryotes species and also present in prokaryotes (Inoue, 1999; Iyer et al., 2008; Koch et al., 2017). MORC superfamily is characterized by a combination of a GHKL-type (gyrase, histidine kinase, and MutL) domain and an S5 domain that constitutes a catalytically active ATPase module (Iyer et al., 2008; Douse et al., 2018). In Human, MORC protein family is represented by MORC1, MORC2, MORC3, and MORC4 displaying common structural determinants such as CW-type zinc finger, and coiled-coil domains. These paralogs appear to have, at least partially, distinct functions as they diverge in tissue-specific expression and contain unique structural motifs. MORC1 was first discovered as a key regulator for male meiosis and spermatogenesis (Inoue, 1999; Pastor et al., 2014). MORC3 was found to play a role in regulating bone and calcium homeostasis (Jadhav et al., 2016). Recently, MORC4 was implicated in S-phase progression during the cell cycle and was associated with acute and chronic pancreatitis, inflammatory disorders, and cancer (Tencer et al., 2020).

*MORC2* gene is expressed in all tissues and MORC2 protein is enriched in the brain (Nagase et al., 1998). The human *MORC2* gene has two isoform transcripts: a long isoform NM_001303256 (5,657 bp) encoding a 1,032 aa protein (NP_001290185) and a short isoform NM_014941 (6,052 bp) encoding a protein of 970 aa (NP_055756). Quantitative RT-PCR on human neural tissue and neuroblastoma cell line SH-SY5Y established that the long isoform is predominantly expressed in neural tissues (Sancho et al., 2019). By convention in this review, all mutations described will refer to the long isoform NM_001303256 which is predominant in the neural tissue. In mouse, two paralogs of *MORC2* are present: *Morc2a* and *Morc2b.* It has been shown that *Morc2b* is restricted to mouse and rat, resulting from a retrotransposition event (Shi et al., 2018). So *Morc2a* gene is referred to as *Morc2* in non-rodent species.

**FIGURE 1:**
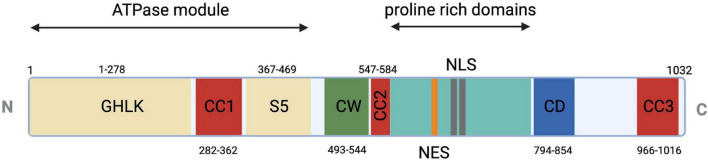
Schematic representation of MORC2 domain architecture. Conserved ATPase module in beige (1–469 a.a.) is a split module composed of GHKL (1–278 a.a.) and S5 (367–469 a.a.) domains. CW-ZF or zinc finger domain is represented (490–544 a.a.) in green and multiple coiled coils in red as CC1 or coiled coil 1 (282–362 a.a.), CC2 or coiled coil 2 (547–584 a.a.), CC3 or coiled coil 3 (966–1016 a.a.). Two unique domains, proline-rich domain (581–788 a.a.) and CD or chromo-like domain (794–854 a.a.), are shown in cyan and blue, respectively. Additional nuclear export signal (691–703 a.a.) and nuclear localization signal (734–742 a.a. and 755–771 a.a.) are shown in orange and gray, respectively.

MORC2 protein includes the characteristic catalytic ATPase module composed of the GHKL-type domain and the S5 domain, three coil-coiled domains allowing dimerization or interaction with protein complexes, a zinc-finger CW domain allowing DNA interaction, and a CHROMO-like (CHRromatin Organization Modifier) domain allowing histone recognition (Figure 1). MORC2 protein contains a nuclear localization signal (NLS) and a nuclear export signal (NES) allowing cytoplasmic and nuclear localization. The NLS appears to predominate over the NES so that MORC2 is mainly localized in the nucleus where it participates to DNA damage repair and transcriptional regulation (Wang et al., 2010). Nevertheless, cytoplasmic MORC2 has been shown to be associated with lipogenesis and adipocyte differentiation (Sánchez-Solana et al., 2014).

**FIGURE 2:**
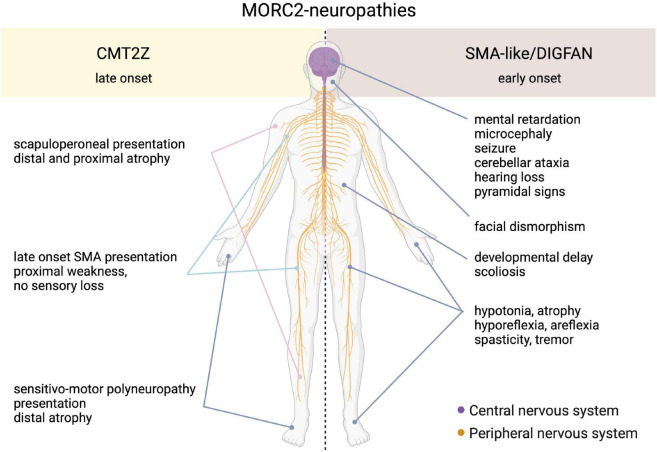
Schematic representation of the clinical features in the spectrum of MORC2-related diseases. On one side, the late onset CMT2Z disease characterized by the peripheral axonopathy with a frequently scapuloperoneal presentation, or more rarely with a sensitive-motor polyneuropathy or a late-onset SMA presentation. On the other side, the early onset SMA-like/DIGFAN syndrome characterized by central and peripheral nervous systems damage, and developmental anomalies.

Heterozygous mutations in the *MORC2* gene have been associated with a spectrum of disorders affecting the peripheral nervous system such as the Charcot-Marie-Tooth disease (CMT) and Spinal Muscular Atrophy-like phenotype disorder (SMA-like), the latter being phenotypically close to a recently described neurodevelopmental syndrome associated with developmental delay, impaired growth, dysmorphic facies, and axonal neuropathy (DIGFAN) (Figure 2).

## Peripheral Nervous System Disorders Associated With MORC2 Mutations

CMT disease refers to a clinically and genetically heterogeneous neuropathy that affects sensory-motor peripheral nerves with an overall prevalence of 1/2,500 (Skre, 1974). The main clinical features of this neuropathy are typically childhood-onset, symmetric weakness and muscle atrophy affecting distal limbs and slowly progressing toward proximal muscles. CMT diseases are classified into two subgroups based on nerve conduction velocity (NCV). CMT1 is characterized by decreased NCV related to myelin sheet degeneration, whereas CMT2 is characterized by normal NCV and decreased amplitude in compound muscular action potential or sensory nerve action potential related to axonal degeneration. Thanks to whole-exome sequencing, more than 80 genes have been linked to different forms of CMT (Neuromuscular Disease Center, Washington University, St. Louis)^1^ with all mode of inheritance: autosomal dominant, autosomal recessive or X-linked. By convention, an alphabetical letter is associated to the CMT subtypes for each new genetic cause identified. For example, CMT1A which represents more than 60% of demyelinating subtypes, is related to mutations in the *PMP-22* gene.

### CMT2Z

In, Sevilla et al. (2016) identified for the first time a rare form of axonal Charcot-Marie-Tooth disease (CMT2Z; OMIM: 616688) related to a mutation in the *MORC2* gene (NM_001303256.3; c.754 C > T; p.R252W) with autosomal dominant segregation in a Spanish family. In this article, MORC2 mutation p.R252W (c.754C > T) was first reported as p.R190W (c.568C > T) based on the short isoform transcript. With these patients, symptoms typically began with cramps in the lower limbs during childhood or early adulthood and evolved into CMT classical features such as lower limb weakness followed by hand weakness, sensory loss, absence of tendon reflex, and *pes cavus*. With the progression of the disease, atypical signs appeared in older patients that presented prominent asymmetric proximal muscle weakness (pelvic and shoulder girdle) but with a relative sparing of knee and elbow extension even in the most severe forms. These observations led some clinicians, in a retrospective analysis of patients diagnosed with CMT2Z, to categorize these CMT2 patients into a new phenotypic group termed the scapuloperoneal phenotype with the aim of ultimately helping clinicians to diagnose patients with a *MORC2* gene mutation (Sivera et al., 2021).

After this first *MORC2* mutation report, clinicians searched for *MORC2* mutations in their genetically undiagnosed CMT2 cohorts and several new cases were identified. Within two years, the point mutation p.R252W associated to similar CMT2 clinical features was found sporadically in two unrelated Australian families (Albulym et al., 2016), a Czech family (Laššuthová et al., 2016), eight unrelated families in Japan (Ando et al., 2017) and in one Korean family (Hyun et al., 2016; Table 1). Additional clinical features were described in some of these patients such as hearing loss (Albulym et al., 2016; Hyun et al., 2016; Sevilla et al., 2016), neck weakness (Hyun et al., 2016; Sevilla et al., 2016), seizure and pyramidal signs (Albulym et al., 2016; Table 2).

**TABLE 1:** MORC2 mutations and reported phenotypes.

Transcript	Protein	Domain	Onset	Diagnostic	Other feature	References
NM_001303256.3	NP_001290185					
c.71C > T	p.T24I	GHKL	Early chilhood	SMA-like / DIGFAN	Developmental delay, facial dismorphism, cortical atrophy, spasticity, hyperreflexia	Guillen Sacoto et al. (2020)
c.79G > A	p.Q27K	GHKL	Early chilhood	SMA-like/DIGFAN	Developmental delay, spasticity, hyperreflexia	Guillen Sacoto et al. (2020)
c.260C > T	p.S87L	GHKL	Early chilhood	SMA-like/DIGFAN	Developmental delay, hearing loss, facial dismorphism, mental retardation	Guillen Sacoto et al. (2020)
c.260C > T	p.S87L	GHKL	Birth	SMA-like/DIGFAN	Cerebellar hypoplasia	Duan et al. (2021)
c.260C > T	p.S87L	GHKL	Birth	SMA-like/DIGFAN	Microcephaly, areflexia	Sevilla et al. (2016)
c.260C > T	p.S87L	GHKL	Birth	SMA-like/DIGFAN	Developmental delay, generalized hypotonia, sensory loss, scoliosis	Hyun et al. (2016)
c.263C > T	p.A88V	GHKL	nd	SMA-like/DIGFAN	Developmental delay, intellectual disability, facial dismorphism, hearing loss, spasticity	Guillen Sacoto et al. (2020)
c.394C > T	p.R132C	GHKL	Childhood	SMA-like/DIGFAN		Guillen Sacoto et al. (2020)
c.395G > T	p.R132L	GHKL	Childhood	CMT2/Scapuloperoneal	Neck weakness, hearing loss	Hyun et al. (2016)
c.455C > G	p.A152P (VUS)	GHKL	Childhood	CMT2/SM polyneuropathy		Sivera et al. (2021)
c.707A > G	p.E236G	GHKL	Childhood	CMT2/Scapuloperoneal	Seizure, wheelchair hypoventilation	Albulym et al. (2016)
c.754 C > T	p.R252W	GHKL	Childhood	CMT2/Scapuloperoneal	Few hearing loss, neck weakness	Sevilla et al. (2016)
c.754 C > T	p.R252W	GHKL	Childhood	CMT2/Scapuloperoneal		Laššuthová et al. (2016)
c.754 C > T	p.R252W	GHKL	Childhood	CMT2/Scapuloperoneal	Few hearing loss, neck weakness, no pyramidal signs	Hyun et al. (2016)
c.754 C > T	p.R252W	GHKL	Childhood	CMT2/Scapuloperoneal	Few hearing loss, pyramidal sign, hearing loss, seizure	Albulym et al. (2016)
c.754 C > T	p.R252W	GHKL	Childhood	CMT2/Scapuloperoneal		Miressi et al. (2020)
c.754C > T	p. R252W	GHKL	Childhood	CMT2/SM polyneuropathy	Axonal motor neuropathy with high variability in disease severity and duration	Duan et al. (2021)
c.798G > C	p.R266S	CC1	Childhood	SMA-like/DIGFAN	Hypotonia, hyporeflexia, hearing loss, scoliosis	Guillen Sacoto et al. (2020)
c.955C > T	p.R319C	CC1	Adulthood	CMT2/SM polyneuropathy		Sivera et al. (2021)
c.956G > A	p.R319H	CC1	Adulthood	SMA late onset		Karakaya et al. (2018)
c.1164C > G	p.S388R	S5	Childhood	SMA-like/DIGFAN	Hypotonia, spasticity, cerebellar atrophy, hearing loss	Guillen Sacoto et al. (2020)
c.1181A > G	p.Y394C	S5	Childhood	SMA-like/DIGFAN	Hypotonia, spasticity	Guillen Sacoto et al. (2020)
c.1181A > G	p.Y394C	S5	Childhood	CMT2/Scapuloperoneal		Ando et al. (2017)
c.1181A > G	p.Y394C	S5	nd	SMA-like/DIGFAN	Developmental delay, intellectual disability	Sivera et al. (2021)
c.1199A > G	p.Q400R	S5	Childhood	CMT2/Scapuloperoneal		Ando et al. (2017)
c.1217C > T	p.A406V	S5	Childhood	CMT2/Scapuloperoneal		Sivera et al. (2021)
c.1220G > A	p.C407Y	S5	Childhood	CMT2/SM polyneuropathy		Ando et al. (2017)
c.1220G > A	p.C407Y	S5	Childhood	CMT2/SM polyneuropathy		Duan et al. (2021)
c.1237G > T	p.V413F	S5	Childhood	SMA-like/DIGFAN	Hypotonia, hyporeflexia, ataxia	Guillen Sacoto et al. (2020)
c.1265A > G	p.E422G	S5	Early chilhood	SMA-like/DIGFAN	Microcephaly, “Leigh syndrome” presentation	Yang et al. (2021)
c.1271C > G	p.T424R	S5	Early chilhood	SMA-like/DIGFAN	Diaphragmatic paralysis, cerebellar atrophy, developmental delay	Schottmann et al. (2016)
c.1271C > G	p.T424R	S5	Early chilhood	SMA-like/DIGFAN	Diaphragmatic paralysis, cerebellar ataxia, facial anomalies, microcephaly, developmental delay	Zanni et al. (2017)
c.1292C > T	p.A431V	S5	Childhood	CMT2/Scapuloperoneal	Brain, spinal cord atrophy, mental retardation	Ando et al. (2017)
c.1330G > A	p.G444R	S5	nd	CMT2/Scapuloperoneal		Albulym et al. (2016)
c.1330G > A	p.G444R	S5	Adulthood	CMT2/Scapuloperoneal		Jacquier et al. (2022)
c.1338C > A	p.H446Q	S5	Adulthood	SMA late-onset		Jacquier et al. (2022)
c.1396 G > C	p.D466N	S5	Chilhood	CMT2/Scapuloperoneal	Scoliosis	Semplicini et al. (2017)
c.1397A > G	p.D466G	S5	Adulthood	CMT2/Scapuloperoneal	HyperCKemia	Duan et al. (2021)
c.1397A > G	p.D466G	S5	Adulthood	CMT2/Scapuloperoneal	Hyperhydrosis, tremor	Sivera et al. (2021)

*Table describe patient main diagnostic, onset and some additional features for each point mutation in MORC2 transcript (NM_001303256.3) and it consequence on MORC2 protein (NP_001290185).*

**TABLE 2:** Summary of clinical characteristics of individuals with MORC2 mutations.

	SMA-like/DIGFAN		CMT2Z	
	Case report	In %	Case report	In %
**Central phenotypes**				
Intellectual disability	23	79.3	12	16.4
Facial dysmorphism	19	65.5	1	1.4
Microcephalia	17	58.6	0	0.0
Hearing loss	13	44.8	4	5.5
Retinopathy	5	17.2	2	2.7
Seizures	2	6.9	3	4.1
Pyramidal feature/hypereflexia	7	6.9	8	11.0
**Peripheral phenotypes**				
Motor developmental delay	27	93.1	9	12.3
Hypotonia	18	62.1	2	2.7
Proximal weakness	22	75.9	57	78.1
Distal weakness	11	37.9	72	98.6
Sensory involvement	4	13.8	59	80.8
Asymetry	0	0.0	4	5.5
Pes cavus	9	31.0	37	50.7
Scoliosis	9	31.0	5	6.8
Total patient reported	29		73	

*Percentage have been calculated on the total of patient reported including non-determined feature patients as negative one. Table describe the percentage of clinical features reported in the literature and diagnosed as a SMA-like/DIGFAN syndrome or as CMT2Z disease. In this retrospective analysis, all the criteria were not available for each patient. Non reported phenotypes were considered as a negative one to calculate the percentage.*

**FIGURE 3:**
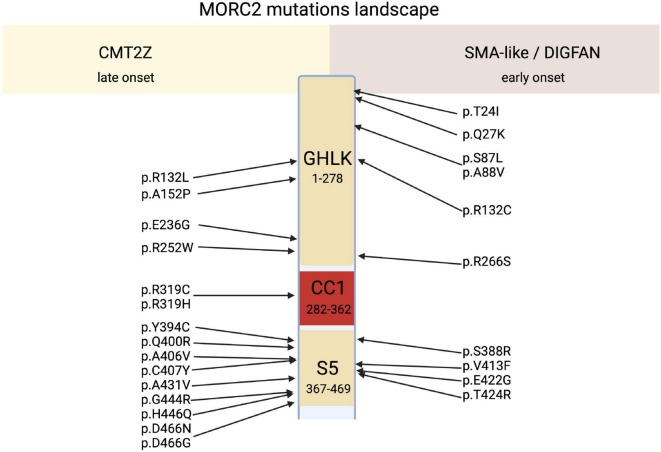
Schematic representation of MORC2 domains architecture correlated to their related main clinical features, CMT2Z or SMA-like/DIGFAN syndrome.

Along with the p.R252W mutation, which is the most frequent mutation, other mutations related to CMT2 were identified all along the GHKL-type and S5 domains (Figure 3). Mutations p.R132L (Hyun et al., 2016), p.E236G (Albulym et al., 2016), p.Y394C (Ando et al., 2017), p.A406V (Sivera et al., 2021), p.G444R (Albulym et al., 2016; Jacquier et al., 2022), p.D466N (Semplicini et al., 2017), p.Q400R (Ando et al., 2017), p.D466G (Duan et al., 2021; Sivera et al., 2021) were associated with a CMT2 scapuloperoneal phenotype, whereas p.R319C (Sivera et al., 2021), p.C407Y (Ando et al., 2017) were associated with a pure sensory-motor polyneuropathy without proximal involvement. This discrepancy in phenotype could be explained by a differential effect of the various mutations on MORC2 activity and/or by differences in the age of the patient at the time of examination. Additionally, a variant of unknown significance p.A152P (Sivera et al., 2021) was reported in CMT2 patients without clear evidence of pathogenicity. Altogether, MORC2 mutations were detected in 2.7% of patients with CMT type 2 in a Japanese cohort (Ando et al., 2017) thus making *MORC2* the second most common gene causative of CMT2 after *MFN2*.

### Spinal Muscular Atrophy-Like, Developmental Delay, Impaired Growth, Dysmorphic Facies, and Axonal Neuropathy Syndrome

MORC2 mutations occasionally produce early onset spinal muscular atrophy-like (SMA-like) phenotypes characterized by primary involvement of proximal muscle atrophy. Using direct sequencing in 52 unrelated probands, a *de novo* heterozygous missense mutation p.S87L was identified in a patient with infantile-onset SMA-like presentation associated with microcephaly, general weakness and areflexia (Sevilla et al., 2016). After this first report, clinicians re-examined sequencing data from patients with genetically unsolved neuropathies for mutations in MORC2 and identified a similar p.S87L mutation in two unrelated patients with infantile-onset SMA-like picture associated with generalized hypotonia, sensory loss, developmental delay, cataract, scoliosis, dysmorphic face (Hyun et al., 2016) or in one patient with scoliosis, cerebellar hypoplasia, and intellectual disability (Duan et al., 2021; Table 1). Besides the p.S87L mutation, patients with similar SMA-like picture were rapidly identified in two unrelated families with a *de novo* p.T424R (c.1271C > G) *MORC2* mutation associated with cerebellar atrophy, diaphragmatic paralysis, developmental delay (Schottmann et al., 2016; Zanni et al., 2017). Similarly, p.A431V and p.E422G mutations were identified in three sporadic SMA-like patients also showing brain atrophy and intellectual disability (Ando et al., 2017; Yang et al., 2021).

Recently, 20 individuals with heterozygous *MORC2* mutations sharing common features such as developmental delay, impaired growth, facial dysmorphism, and axonal neuropathy were identified in large cohorts of patients with neurodevelopmental disorders. This defined a new neurodevelopmental syndrome called DIGFAN (OMIM: 619090) (Guillen Sacoto et al., 2020). This study allowed the identification of several new MORC2 mutations such as p.T24I, p.Q27K, p.A88V, p.R256C, p.S388R, p.V413F, and also identified new patients carrying the already known mutations p.S87L and p.Y394C initially described as causing SMA-like features. In fact, retrospective analysis of the early onset SMA-like patients shows similar clinical features with the DIGFAN neurodevelopmental syndrome and should be considered as a single clinical entity.

### Adult-Onset Spinal Muscular Atrophy

Interestingly, one patient suspected of having developed an adult-onset SMA without *SMN1* mutation was finally classified as CMT2Z after the identification of p.R319H mutation in *MORC2* (Karakaya et al., 2018). Recently, a second patient with a clinical presentation of late-onset SMA was identified in a French family with the heterozygous p.H446Q mutation, suggesting that genetically unsolved late-onset SMA patients should be considered for *MORC2* sequencing (Jacquier et al., 2022).

Altogether, these case reports allow to make some initial statements: (i) MORC2 mutations are associated with a spectrum of neuropathies; (ii) all the mutations are localized in the catalytic domain of the protein, suggesting that MORC2 ATPase function is important; (iii) some mutations cause the same clinical feature in the different individuals, although the degree of severity remains variable. This indicates that genotype-phenotype correlations can be established; (iv) The understanding of MORC2 biological functions could shed light on the pathophysiology of these disorders.

## Molecular Functions of MORC2

### Transcriptional Regulation

MORC2 has a CW-type zinc finger domain that is present in many chromatin-associated factors (Perry, 2003), and allows interaction with DNA and proteins. With *AtMORC1* and *AtMORC6* homologs in Arabidopsis being involved in transcriptional repression (Moissiard et al., 2012), the role of MORC2 in transcriptional regulation was quickly investigated.

It was demonstrated that, when fused to the Gal4 DNA binding domain, the NLS (aa 734–742 and aa 755–771) and the proline-rich domains (aa 581–788) of MORC2 decrease the expression of a Gal4 reporter transgene (Wang et al., 2010). This suggests that the nuclear localization of MORC2 is associated with transcriptional repression. This hypothesis is supported by MORC2 ability to interact with chromatin modifiers that remove or deposit chromatin marks associated with transcriptional repression. Hence, MORC2 is able to recruit the histone deacetylase 4 (HDAC4) on the carbonic anhydrase IX gene promoter resulting in its transcriptional repression through histone H3 deacetylation (Shao et al., 2010). MORC2 can also recruit the histone deacetylase 1 (HDAC1) on the *p21* gene promoter resulting in *p21* repression (Zhang et al., 2015).

In addition, MORC2 can affect the tri-methylation of histone 3 lysine 9 (H3K9me3) *via* its recruitment to heterochromatic sites by the human silencing hub complex (HUSH) (Tchasovnikarova et al., 2017), as well as the tri-methylation of histone 3 lysine 27 (H3K27me3) by recruiting the polycomb repressive complex 2 (PRC2) to the *ArgBP2* enhancer (Tong et al., 2018).

**FIGURE 4:**
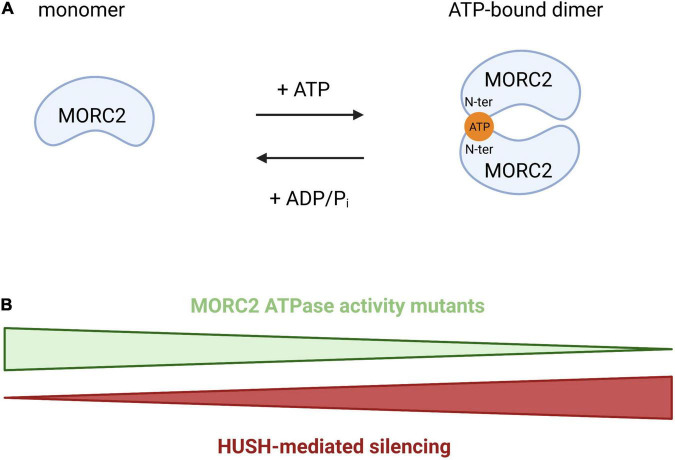
Schematic representation of MORC2 homodimerization through ATP binding. **(A)** Model proposed by Douse et al. (2018) where the N-terminal part of MORC2 can homodimerize through ATP binding, regulating HUSH-mediated repression. **(B)** An increase of the ATP hydrolysis will favor the monomeric form of MORC2 leading to a loss of HUSH-mediated silencing and vice-versa.

Initially discovered for its involvement in the repression of active genes integrated into heterochromatin (a phenomenon called position effect variegation), the HUSH complex is composed of three proteins: Transgene Activation suppressor (TASOR), M-Phase Phosphoprotein 8 (MPP8), and Periphilin (PPHLN1) (Tchasovnikarova et al., 2015). HUSH is able to recognize the H3K9me3 chromatin mark thanks to the chromodomain of MPP8. Coupled with this mechanism, it was recently demonstrated that the HUSH complex has evolved to recognize long intronless transcription units, a main feature of retroelements (Seczynska et al., 2022). In this context, HUSH spreads H3K9me3 *via* the recruitment of the methyltransferase SET Domain Bifurcate 1 (SETDB1) to repress transposable mobile elements, which are potential threats for genomic integrity (Liu et al., 2017; Robbez-Masson et al., 2018), but it also participates to the regulation of gene expression at heterochromatic loci (Tchasovnikarova et al., 2015). These functions are also regulated by MORC2 since its ATPase activity is required for the activity of the HUSH complex. The N-terminal part of MORC2 can homodimerize upon ATP binding and this homodimerization is required for HUSH activity. So, an increase in ATP hydrolysis will favor the monomeric form of MORC2 leading to a loss of HUSH-mediated silencing and vice-versa (Figure 4). The efficiency of HUSH-mediated silencing is therefore directly regulated by the ATPase activity of MORC2. So MORC2 is involved not only in the control of the expression of certain genes but also in the maintenance of genomic integrity *via* the HUSH complex.

### DNA Repair

The eukaryotic genome is structured in chromatin. Chromatin is a complex nucleoprotein structure, whose basic unit, the nucleosome is composed of an octamer of histones comprising two copies of each core histone H2A, H2B, H3, and H4, around which 147 bp of DNA are wrapped (Luger, 1997). This structure prevents direct access to the DNA. Therefore, when DNA damage occurs, chromatin must be partially remodeled to facilitate access to repair factors. ATP-dependent chromatin remodelers act in this process to increase DNA accessibility through ATP hydrolysis. Since MORC2 has an ATPase activity, many studies have focused on its role in the response to DNA damage.

**FIGURE 5:**
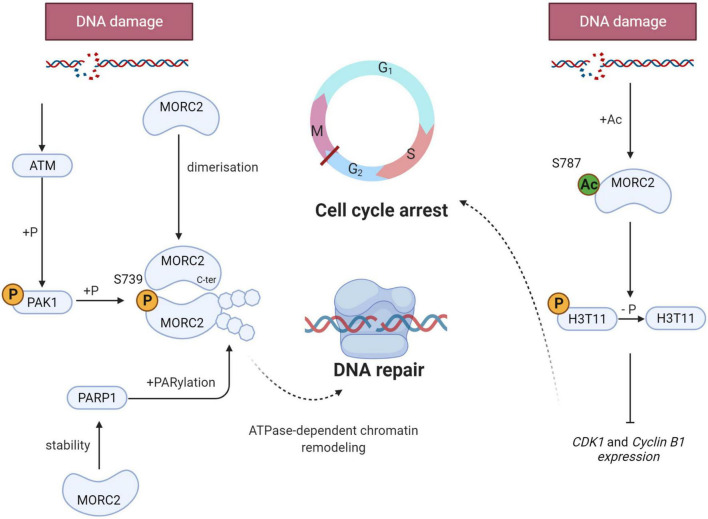
Schematic representation of the key role of MORC2 in the DNA Damage Response (DDR). When DNA damage occurs, MORC2 undergoes several post translational modifications (on the left side) : Serine 739 phosphorylation through ATM/PAK1 pathway, dimerization and PARylation. All modifications allow ATPase-dependent chromatin remodeling and thus enable efficient DNA damage repair. MORC2 can also be acetylated on serine 787 favoring its interaction with the phosphorylation of histone H3 threonine 11 (H3T11P). This will lead to H3T11P dephosphorylation and CDK1 and cyclin B1 repression. G2/M checkpoint will be activated and promotes cell cycle arrest.

When DNA damage occurs, a cellular response, called DNA Damage Response (DDR), is initiated to signal the damage, stop the cell cycle, activate repair mechanisms, and eliminate cells if the genome is irreversibly damaged. DDR is characterized by the phosphorylation of hundreds of proteins. Ataxia telangiectasia mutated (ATM) is one of the most upstream kinases in this pathway. Among ATM substrates, p21 activated kinase (PAK1) is phosphorylated in response to ionizing damage (Li et al., 2012). This serves to activate its kinase activity leading to the phosphorylation of MORC2 on serine 739, promoting its association with chromatin and its ATPase activity to induce chromatin relaxation (Figure 5). MORC2 also induces the phosphorylation of H2AX (γH2AX), a key mediator for an efficient repair process. In addition, MORC2 is able to homodimerize through its C-ter coiled-coil domain (Xie et al., 2019). MORC2 dimerization is necessary for its chromatin remodeling function and for the induction of γH2AX, allowing the recruitment of key repair factors such as Breast Cancer 1 (BRCA1) or p-53 binding protein 1 (53BP1).

In parallel to ATM-mediated phosphorylation, DDR is also characterized by the recruitment of poly(ADP-ribose) polymerase 1 (PARP1) at damaged sites. This enzyme synthesize PAR polymers that can post-translationally modify different proteins, also referred to as parylation. Histone parylation allows the relaxation of chromatin, and consequently the access to DNA strand breaks, facilitating the recruitment of PAR-interacting DNA repair proteins to damaged sites. It has been evidenced that PARP1 enables the recruitment of MORC2 to DNA lesions and induces its parylation to facilitate MORC2 chromatin remodeling activity (Figure 5). Interestingly, it has been shown that MORC2 increases the stability of PARP1, inducing a positive feedback loop between the two factors to enable efficient DNA damage repair (Zhang and Li, 2019).

DDR also activates cell cycle checkpoints to block cell cycle progression to facilitate the repair process (Visconti et al., 2016). The G2/M checkpoint prevents cells with unrepaired genomes from entering mitosis and therefore prevents mitotic catastrophes and cell death. At the molecular level, the phosphorylation of histone H3 threonine 11 (H3T11P) is decreased as a result of DNA damage, which induces the transcriptional repression of *cyclin dependent kinase 1 (CDK1)* and *cyclin B1* genes, that code for two key regulators of the G2/M transition required for entry into mitosis (Shimada et al., 2008). Upon DNA damage, MORC2 is acetylated on lysine 787, which favors its interaction with H3T11P and contributes to H3T11P dephosphorylation, leading to the decrease of CDK1 and cyclin B1 (Liu et al., 2020; Figure 5). All these studies demonstrate that MORC2 is a key factor in DDR, both through its chromatin remodeling activity and its control of cell cycle checkpoints.

### Metabolism

**FIGURE 6:**
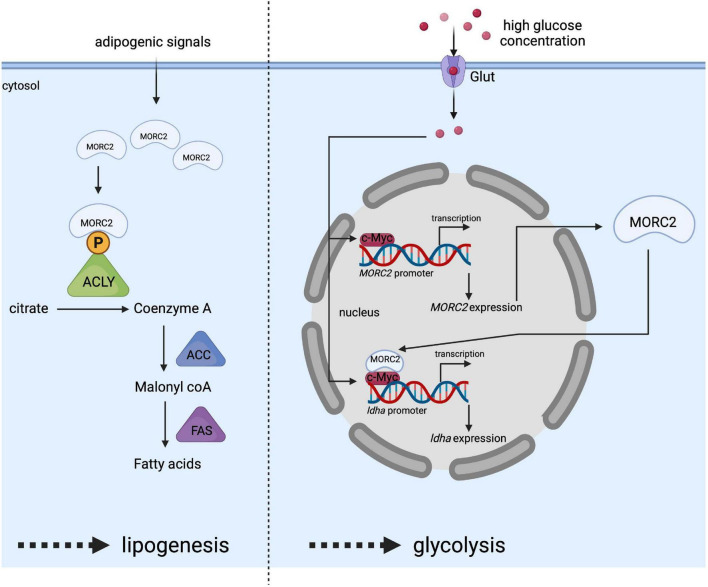
Schematic representation of MORC2 as a key regulator of lipogenesis (left side) and glycolysis (right side) pathway. Adipogenic signals increase MORC2 protein levels facilitating the interaction between MORC2 and ACLY in the cytosol, and leading to the activation of ACLY by preventing its dephosphorylation. This activated ACLY is essential for increased fatty acid synthesis. High glucose concentration in the culture medium stimulates the induction of *MORC2* expression. Once, MORC2 is induced, it forms a complex with c-myc and gets recruited to *LDHA* promoter to induce *LDHA* expression, which in turn facilitates glycolysis.

ATP citrate lysase (ACLY) is involved in the early steps of lipogenesis since it allows the synthesis of acetyl-CoA, an essential metabolite. ACLY has been identified as a potential MORC2 protein interactor *via* immunoprecipitation/mass spectrometry (Sánchez-Solana et al., 2014). The interaction takes place specifically in the cytoplasm and increases the available pool of ACLY. It has been proposed that MORC2 binding would prevent ACLY dephosphorylation, thereby increasing its stability and enzymatic activity (Figure 6). MORC2 would therefore be an important player in controlling the cellular level of available acetyl CoA. MORC2 also regulates the protein level of all lipogenesis proteins such as acetyl CoA carboxylase (ACC) and fatty acid synthase (FAS), making MORC2 a major regulator of fatty acid biosynthesis. In addition, acetyl CoA is also the substrate of the mevalonate pathway (Zaidi et al., 2012) to be later transformed into cholesterol or isoprenoids. The authors therefore focused on the role of MORC2 in this pathway and determined that the mRNA level of 3-hydroxy-3-methylglutaryl-CoA reductase (HMGCR) is decreased in the absence of MORC2. This enzyme is located upstream of the mevalonate pathway and is required for its completion (Davidson, 2001). This suggests that MORC2 also probably indirectly regulates the mevalonate pathway. Since lipogenesis is important for adipogenesis, it has been suggested that MORC2 regulates this process following the induction of its expression during adipogenic differentiation (Sánchez-Solana et al., 2014). This mechanism has been confirmed *in vivo* in mice during mammary gland development where an increase in MORC2 expression coincides with the onset of lipogenesis and ACLY activity during pregnancy (Sánchez-Solana et al., 2014).

The involvement of MORC2 does not seem to be limited to lipogenesis and the mevalonate pathway, and a recent study indicated that MORC2 regulates glucose metabolism (Guddeti et al., 2021). Binding of the transcription factor c-myc to the MORC2 promoter stimulates its expression in response to high concentrations of glucose making MORC2 expression glucose-inducible (Figure 6). MORC2 expression is positively correlated with the expression of enzymes involved in glycolysis [e.g., hexokinase 1, lactate dehydrogenase A (LDHA), phosphofructokinase liver type (PFKL), and phosphofructokinase platelet isoform (PFKP)]. Specifically for LDHA, reciprocal cooperativity was described between MORC2 and c-myc, consisting in the stimulation of *MORC2* gene expression by c-myc and in the association of newly synthesized MORC2 with c-myc to induce *LDHA* gene expression (Figure 6).

All these data indicate that MORC2 plays key roles in different metabolic pathways. It is interesting to notice that MORC2 dysregulation favors the appearance of pathologies associated with metabolic disorders. For example, a perturbation of MORC2 localization disrupts adipocyte homeostasis in mice (Hallenborg et al., 2021), leading to an increase in adipose tissue mass, glucose intolerance and hepatic steatosis in young mice. On the other hand, an increase in MORC2 expression promotes the growth of cholangiocarcinoma associated with increased glutamine metabolism (Su et al., 2021).

## Pathophysiological Mechanisms in Neurons

The previous sections described the ability of MORC2 to homodimerize either *via* its N-terminally located ATPase module to regulate HUSH-mediated silencing (Douse et al., 2018) or *via* its C-terminal coiled-coil domain for the DDR (Xie et al., 2019). This may explain the dominant-negative character of MORC2-related neuropathic mutations, at least for these two pathways. It is worth noticing that according to the International Mouse Phenotyping Consortium, heterozygous *Morc2a* Knock-out (KO) mice display abnormal gait and decreased levels of circulating high density lipoproteins (HDL) cholesterol.^2^ These results suggest that haploinsufficiency should also be taken into consideration in the dominant nature of MORC2 mutations. Although MORC2 is expressed in all tissues, MORC2 mutations preferentially impact the nervous and muscular systems. What could be the rational?

In humans, CMT2Z first affects the peripheral nerves and then progresses to other structures (Sevilla et al., 2016). The muscle decay observed in patients would be primarily due to an impairment of the motor nervous system resulting in muscle atrophy due to a lack of stimulation. Hence, we will hereafter focus mainly on the mechanisms that can explain the specific damage to neurons, considering that the effect on muscles is secondary to the effect on the nervous system. Nevertheless, given the similarities between neuron and muscle (post-mitotic electrically active cells with high metabolic rates), we cannot exclude that the mechanisms described below also happen in muscle.

**FIGURE 7:**
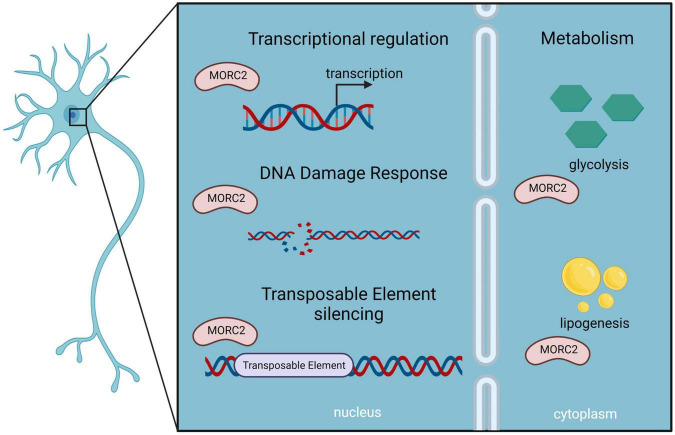
Schematic representation of MORC2 functions as described in neurons, which could be implicated in MORC2-related diseases.

In mouse, *Morc2* expression is dynamic during development with a peak of expression in early phases followed by a decrease during aging (Sancho et al., 2019). *Morc2* is highly expressed in the brain, exclusively in neurons, and a *Morc2* KO in mice is lethal at embryonic day 13.5 (Hagelkruys et al., 2021). Altogether, these observations suggest a critical role of MORC2 during development especially in the brain. MORC2 has being involved in transcriptional regulation, DNA damage, and fatty acid metabolism, this section explores the possible pathological mechanisms caused by a dysregulation of each of its pathways (Figure 7).

### Transcriptional Regulation

MORC2 is able to interact with several proteins to regulate gene expression, including HDAC4 (see above). HDAC4 has been involved in multiple aspects of neuronal functions (Fitzsimons, 2015). Intriguingly, a heterozygous mutation in *HDAC4* has been linked to chromosome 2q37 deletion syndrome (OMIM 600430) (Williams et al., 2010) characterized by impaired cognition, which is also observed in DIGFAN patients. Furthermore, HDAC4 overexpression has been identified in the muscle of SMA patients (Bricceno et al., 2012), and as explained above MORC2 is implicated in SMA-like phenotype. HDAC1 has been involved in neuronal development (Jaworska et al., 2015). Like HDAC4, MORC2 interacts with HDAC1 to regulate the expression of specific genes. Further studies are thus needed to understand the role of MORC2 in the neurogenetic function of HDAC4 and HDAC1.

MORC2 is also involved in transcriptional repression through H3K9me3 deposition mediated by the HUSH complex. Numerous studies have established the role of MORC2 in controlling the expression of, among others, transposable elements (TEs) (Liu et al., 2017), Kruppel-associated box zinc finger (KZNF) genes (Tchasovnikarova et al., 2015) and more recently the protocadherin cluster (Hagelkruys et al., 2021). Interestingly, all three types of loci have been reported to be independently involved in neurogenesis. TEs are DNA sequences that have retained their ability to move in the genome by transposition. The mechanism allowing transposition is often imprecise resulting in potential alteration of neighboring host sequences and misregulation of gene expression (Bourque et al., 2018). TEs are the main sources of the emergence and evolution of long non-coding RNAs (lncRNAs). For example, in humans, 13,000 lncRNAs have been identified. Human endogenous retroviruses (HERVs) insertions are responsible for their transcription by providing promoter sequences. LncRNAs can serve as epigenetic regulators, some of them implicated in neurogenesis *via* their participation in the establishment of a specific transcriptional program (Mustafin and Khusnutdinova, 2020). TEs can also be a source of genetic instability. To counteract this phenomenon, KZNFs transcription factors have co-evolved to acquire the ability to target TEs to recruit KRAB associated protein 1 (KAP1). KAP1 serves as a scaffold for other chromatin modifiers to enable transcriptional repression (Rowe et al., 2010). It was recently proposed that the action of KZNFs is not limited to recruit KAP1, but can form an additional layer of epigenetic regulation in combination with TEs. For example, ZNF417/587 regulate specific brain and spinal cord enhancers that regulate neuronal differentiation and neurotransmission. They also prevent the synthesis of retroviral envelope proteins that are particularly involved in neurodegeneration (Turelli et al., 2020). Finally, in a *Morc2* KO mouse model, the overexpression of the protocadherin cluster has been associated with a loss of neuromuscular functions (Hagelkruys et al., 2021). In the same study, a *Mpp8-Morc2* double KO mouse model was shown to develop the same phenotype than the *Morc2* KO and the *Mpp8* KO mice, suggesting that a common pathway (the HUSH complex) is responsible for the phenotype. Protocadherins are involved in the discrimination of self vs. non-self. The protocadherin genes are organized in clusters, each of them with its own promoter, allowing the establishment of a unique barcode for each neuron, and guiding the interactions between neurons (Wu et al., 2001). By preventing the neurites of a single neuron from connecting back to the same neuron, protocadherins promote both the diversity and complexity of the neuronal circuits (Lefebvre et al., 2012). Altogether, these data suggest that MORC2 is a key transcriptional regulator in neurons and brain development. A transcriptome study on rat sensory neurons expressing the MORC2 p.R252W or p.S87L mutants, showed that neurons displayed transcriptional dysregulation of the ZNF-family, of neurotransmitters, and of the kinesin family genes compared to controls (Sancho et al., 2019). Interestingly, the study also identified the downregulation of the heat shock protein Hspb1, also called HSP27. HSP27 is a ubiquitin-binding protein involved in proteasomal degradation of certain proteins under stress conditions, and mutations in its gene is known to cause the autosomal dominant CMT type 2F (CMT2F; OMIM 606595). As such, this study suggested that MORC2-related neuropathic mutations may alter its role in the transcriptional regulation of important genes for neuronal maintenance.

### DNA Damage

Neurons are post-mitotic cells derived from neural stem cells during central nervous system development and must remain functional throughout an individual’s lifetime as most of them cannot be replaced. The replacement of neurons by neural stem cells has been identified in the central nervous system of rodents, but remains controversial in adult humans (Sorrells et al., 2018; Nicaise et al., 2020). Furthermore, like all cells in the body, neurons experience DNA damage on a daily basis (Jackson and Bartek, 2009). Due to their high metabolic activity, neurons generate high levels of free radicals that favor the appearance and accumulation of oxidative DNA damage. Proper management of DNA damage is thus particularly important in this cell type. Hence, defects in DNA repair have been associated with both developmental and age-associated neurodegenerative diseases (Chow and Herrup, 2015). One study suggested a direct link between DNA damage and the development of CMT. Using a genome-wide siRNA screen to identify regulators of DDR, the study demonstrated the enrichment of many genes known to be involved in CMT (Paulsen et al., 2009). Mouse *Morc2* and human *MORC2* genes exhibit ∼97% amino acid similarity in the ATPase domain. Using CRISPR/Cas9-mediated genome editing, a recent study has developed a *Morc2a* c.C260T knock-in mouse model which leads to the generation of heterozygous *Morc2* p.S87L mice (Lee et al., 2021). This mouse model develops axonal neuropathy, locomotor dysfunction, skeletal muscle weakness but also cerebellar ataxia and motor neuron degeneration, thus recapitulating the features of central and peripheral neuropathies associated with MORC2 mutations in humans. At the molecular level, both peripheral and central nervous system neurons display an accumulation of DNA damage coupled with neuronal apoptosis (Lee et al., 2021). This could partially explain the peripheral neuropathy while highlighting the possible impact on the CNS leading to DIGFAN syndrome. The p.T424R mutation is the second mutation associated with DIGFAN, and like p.S87L it strongly deregulates the ATPase activity of MORC2 (Douse et al., 2018). Therefore, it would be interesting to investigate the effect of this mutation on DNA damage-induced neuronal death. Interestingly, MORC2 mutations involved in CMT2Z are also localized in the ATPase module, and some of them disrupt its function (Douse et al., 2018). Given the crucial role of the ATPase-dependent chromatin remodeler activity of MORC2 in DDR, future studies will be needed to understand the impact of these mutations in DDR and neuronal survival.

A recent study provided evidence that DNA damage repair would not occur randomly in the neuronal genome (Reid et al., 2021). The study showed that neurons contain DNA repair hotspots that favor the repair of genes that are essential for proper neuronal function (Reid et al., 2021). Similarly, certain chromatin loci would preferentially accumulate DNA damage in neurons. These include enhancers, which are crucial for the regulation of gene expression (Wu et al., 2021). Hence, the authors propose that DNA damage and repair could be a programmed process designed to open up enhancers and facilitate transcription. This hypothesis fits with a previous study indicating that neuronal activity induces DNA double strand breaks that correlate with the expression of genes involved in learning and memory (Madabhushi et al., 2015). Thus, neurons appear to have evolved to exploit the DDR to ensure longevity but also to enable specific transcriptional regulations. If the DDR is deregulated by MORC2 mutants, an accumulation of mutations could therefore occur at essential genes as well as in enhancers. It is noticeable that PARP1 is an essential actor of DDR and that its stability depends on MORC2 activity (Zhang and Li, 2019). Together, these data suggest that a dysregulation of a MORC2-associated DDR process may promote the development of neuropathic diseases through dysregulation of the neuron-specific transcriptional program leading to neuronal dysfunction or death.

These results fit with other genetic data showing that DDR dysregulation leads to neurologic alteration-associated diseases, such as: Ataxia-telangiectasia, caused by ATM mutations (Amirifar et al., 2020); Xeroderma Pigmentosum, caused by mutations in several genes involved in the nucleotide excision repair pathway (Uribe-Bojanini et al., 2017); or Ataxia with oculomotor Apraxia Type 1, caused by Aprataxin (APTX) mutation involved in single-strand break DNA repair (Coutinho et al., 1993).

### Metabolism

In the nervous system, lipid homeostasis is particularly important since, among other processes, it regulates synaptogenesis and myelin formation, thus regulating electrical currents in axons. Therefore, alterations in lipogenesis could induce neurodegeneration. A recent study indicates that the multienzyme fatty acid synthase (FAS) which converts glucose into fatty acids, is required for brain development (Gonzalez-Bohorquez et al., 2022). A loss of FAS causes microcephaly and is associated with developmental epileptic encephalopathy, two phenotypes also observed in some patients with MORC2-related neuropathies. Interestingly, an alteration of lipid metabolism is also present in CMT2B related to mutations in *RAB7A* that codes for a small GTPase involved in late endocytic trafficking (Giudetti et al., 2020). The authors proposed that these mutations disrupt the autophagic machinery which may induce lipid accumulation that is a possible source of neurodegeneration (Welte, 2015). Therefore a decrease in lipid anabolism (loss of FAS) or lipid accumulation in CMT2B both result in neuronal damage.

Cytoplasmic MORC2 also interacts with ACLY and promotes its ability to catalyzes the formation of acetyl-CoA (Sánchez-Solana et al., 2014). Acetyl-CoA is at the node of several pathways from carbohydrate and lipid metabolism to protein acetylation (Shi and Tu, 2015). Acetyl-CoA is a substrate for choline acetyl transferase that allows the synthesis of acetylcholine, the neurotransmitter used at the neuromuscular junction. Hence, acetylcholine and acetyl-CoA are important molecules for motoneuron function and survival. The regulation of acetyl-CoA availability by MORC2 can thus be paramount for neuronal survival. Given the major role of MORC2 in the regulation of the fatty acid pathway, future studies are required to investigate whether MORC2 mutations disrupt lipid homeostasis in patient neurons.

## Phenotypic Variability

The question remains on how to explain the vast diversity of phenotypes in CMT2Z patients?

### Different Mutations Produce Different Phenotypes

A simple and straightforward answer would be that each mutation in MORC2 ATPase activity leads to a specific dysregulation. Indeed, although all known mutations are localized in the ATPase module, the consequences on MORC2 ATPase activity are heterogeneous. The two most frequent mutations found in SMA-like/DIGFAN syndrome were reported to have opposite effects (Douse et al., 2018). The latter study concluded that the p.T424R mutation would increase ATP hydrolysis whereas the p.S87L mutation would decrease it. Interpretation of the effect of the p.R252W mutation, the most frequent in CMT2Z, seems, however, more subtle. An initial study established that the mutation decreased ATP hydrolysis (Douse et al., 2018), while another study found no alteration of ATP hydrolysis (Sancho et al., 2019). This discrepancy in conclusions could be explained by the difference in the approaches used. The Sancho et al. (2019) study used nuclear extracts of transfected HeLa cells expressing different MORC2 constructs, whereas Douse et al. (2018) used a purified N-terminal portion of MORC2 in an *in vitro* approach. As such, this study analyzed the biological consequences on a truncated protein, and as mentioned by the authors, this test could miss the autosomal dominant character of the pathology by studying the homodimerization of two mutated forms of MORC2.

p.R252W, p.S87L, p.T424R are the three mutations for which the ATPase activity of MORC2 has been evaluated. In the previous paragraphs, we emphasized the importance of the ATPase activity of MORC2 in mediating its functions in DDR and in the regulation of the HUSH complex. DDR and HUSH are both involved in mechanisms necessary for the proper functioning and survival of neurons. It would then be relevant to design experiments to analyze the effect of all the mutations affecting the ATPase activity of MORC2, taking into account the autosomal dominant character of the disease. This would be particularly informative since Douse et al. (2018) proposed that MORC2 ATPase activity directly regulates HUSH-mediated repression (see above). Using a reporter coupled with a rescue approach, Douse et al. (2018) were able to demonstrate that both the p.R252W and p.S87L mutations induce hyperactivation of the HUSH complex. Guillen Sacoto et al. (2020) later evidenced that all mutations found in DIGFAN patients hyperactivate the HUSH complex, although to various degrees. Such activation of the HUSH complex could result in uneven repression of transposable elements, KZNFs transcription factors, and of the protocadherin cluster. The impact on the transcriptome could then be different from one mutation to another, depending on the level of activation of HUSH. The study by Sancho et al. (2019) fits with this aspect, as it highlights differences in the transcriptome from rat sensory neurons transduced with p.S87L and p.R252W mutated MORC2. It is important to note that until now, all but one neuropathic MORC2 mutation for which the activity of the HUSH complex was evaluated induce HUSH hyperactivation. Only the p.T424R mutation would lead to a hypoactivation of the HUSH complex (Douse et al., 2018). Importantly, the Douse et al. (2018) study did not take into account the dominant character of MORC2-related neuropathies since an exogenous version was provided in a *MORC2* KO background. The results therefore reflect the effect of a homodimerization of two mutated versions of MORC2 on the activity of the HUSH complex, which could exacerbate the phenotype. Due to the autosomal dominant nature of CMT2Z it would thus be interesting (1) to set up a new ATPase and HUSH activity test taking into account this aspect, (2) to test all MORC2 mutations and confirm the p.T424R hypoactivation nature.

### Given Mutations Can Induce Different Phenotypes

We first tried to explain how two different mutations can lead to different outcomes. However, MORC2-related neuropathies seem more complex since the same mutation, such as p.Y394C, can generate different outcomes in patients. To understand the underlying reasons for this discrepancy, we probably first need to understand what differentiates two individuals at the genomic level. The post-genomic era has established that a plethora of differences exists in the genomes of two individuals, including single nucleotide variants, copy number variants and retrotransposon insertions. It is now well established that retrotransposons are active in the brain and contribute to the fact that two cells of the same individual have different genetic makeup (somatic mosaicism). Retrotransposition can occur throughout the life of an individual from neurogenesis (Muotri et al., 2005) to post mitotic neurons (Macia et al., 2017). It appears that the LINE-1 (L1) retrotransposons are particularly mobilized in this process (Macia et al., 2017). Liu et al. (2017) revealed that L1 repression is mediated by the HUSH complex and MORC2. HUSH and MORC2 selectively bind full-length L1s recently located in introns of transcriptionally active genes. By favoring the deposition of the constitutive heterochromatin mark H3K9me3, HUSH, and MORC2 then repress the transcription of these genes. Although this repression is modest it occurs on more than a hundred genes, subsequently possibly affecting the entire transcriptional program of the host cell. A study of the transposable L1 elements on single neurons from human brains found that somatic L1 insertion is not a major generator of genomic neuronal diversity with less than 0.6 unique somatic insertions per neuron (Evrony et al., 2012). Nevertheless, it would be interesting to compare the position of these elements between two patients with the same mutation but harboring different phenotypes. This could explain the dysregulation of some genes in favor of others, explaining the development of a particular phenotype. Moreover, CMT2Z disease may be part of multilocus genetic pathologies (Miressi et al., 2020). In this study, the authors demonstrated that a combination of multiple genomic mutations is responsible for various severities in intra-familial members. In that case, even if the p.R252W mutations were present among individuals, the other genomic variations, mitofusin-2 p.R468H and *Alanyl-TRNA synthetase 1* duplication, found in one of the individuals acted synergically to worthen the phenotype. These data raise the importance of combining genetic analysis tools to better diagnose and understand the correlation between genomic modifications and phenotypic manifestations.

In addition, analysis of the largest family with the p.D466N mutation showed variable phenotypes, ranging from rapidly progressing early onset (first decade) to slowly progressing late-onset (even fourth decade) (Semplicini et al., 2017). This intra-familial variability was associated with a potential gender effect with minoration of the symptoms in females. The exact molecular mechanism behind the differential phenotypes is not known. Interestingly, it was recently shown that in breast cancer cells, estrogens upregulate MORC2 protein level by inhibiting MORC2 autophagic degradation (Yang et al., 2020). Thus, it is tempting to speculate that estrogens may play a positive role in intra-familiar variability by stabilizing the MORC2wt protein over the p.D466N mutant in females. Like this, increased stability of MORC2wt in females could compensate the negative effects mediated by the p.D466N mutation, for example by increasing the ratio of MORC2wt homodimers, explaining the delayed onset and slower progression of the pathology.

## Conclusion

The *MORC2* gene encodes a protein enriched in the brain that plays a key role in chromatin remodeling and epigenetic regulation. MORC2 is implicated in DNA repair, silencing of transposable elements together with the HUSH complex. It is also involved in the regulation of transcription, lipogenesis and glucose metabolism. In neurons, all these functions are essential to allow neurons proper homeostasis, including the long term maintenance of genomic stability. Heterozygous mutations in *MORC2* gene have been associated to a spectrum of peripheral neuropathies from the severe early onset DIGFAN syndrome and SMA-like presentation, to the late onset CMT2Z disease. Interestingly, all MORC2 mutations causing neuropathies are localized in its ATPase catalytic domain and consequently are likely to impact MORC2 ATPase activity, which is essential for its functions. MORC2 homodimerization coupled with a potential haploinsufficiency could explain the dominant negative impact of the mutations. Likewise, the most frequent MORC2 mutation p.R252W is always associated to CMT2Z phenotype while the p.S87L or p.T424R are always associated to the severe DIGFAN syndrome, thus drawing a genotype—phenotype correlation, although the degree of severity varies between individuals. It is interesting to notice that both the p.T424R and p.S87L mutations disrupt the dimerization of the ATPase module, resulting in a strong dysregulation of MORC2 ATPase activity. These two mutations are therefore likely to have the greatest impact on MORC2 function that involve its ATPase activity, especially during development. This could explain why these two mutations are associated with DIGFAN syndrome. It seems imperative to test each known mutation for its impact on MORC2 ATPase activity while taking into account the autosomal dominant character of the disease. Although the mouse model of Lee et al. (2021) is an initial and important step, new animal models are required to cover the variability of MORC2-related neuropathic mutations. On the other hand, the variability of genomic insertion of transposable elements among individuals, the combination of multiple genomic mutations and the gender could also influence the severity and the onset of the disease. Therefore, further investigations supported by next-generation sequencing technologies will certainly improve the understanding of the pathophysiology of MORC2-related neuropathies.

## Author Contributions

AJ and SR conducted the literature search, wrote the first draft of the manuscript, and designed the figures. AJ designed the tables. All authors contributed to conceptualizing, reviewing, editing the manuscript, and approved the submission of the manuscript.

## Conflict of Interest

The authors declare that the research was conducted in the absence of any commercial or financial relationships that could be construed as a potential conflict of interest.

## Publisher’s Note

All claims expressed in this article are solely those of the authors and do not necessarily represent those of their affiliated organizations, or those of the publisher, the editors and the reviewers. Any product that may be evaluated in this article, or claim that may be made by its manufacturer, is not guaranteed or endorsed by the publisher.

## Footnotes

1
http://neuromuscular.wustl.edu/

2
https://www.mousephenotype.org/data/genes/MGI:1921772#phenotypes-section

## Abbreviations

ACLY: ATP citrate lysase
ATM: Ataxia telangiectasia mutated
CDK1: cyclin dependent kinase 1
CMT: Charcot-Marie-Tooth
DDR: DNA Damage Response
DIGFAN: developmental delay, impaired growth, dysmorphic facies, and axonal neuropathy
GHL-type ATPase: Gyrase, Histidine kinase and MutL – type ATPase
LDHA: lactate dehydrogenase A
MORC2: Microrchidia CW-type zinc finger 2
NCV: nerve conduction velocity
NES: nuclear export signal
NLS: nuclear localization signal
SMA-like: Spinal Muscular Atrophy-like
TE: transposable element.
